# Neuropsychological benefits of a narrative cognitive training program for people living with dementia: A pilot study

**DOI:** 10.1590/S1980-5764-2016DN1002008

**Published:** 2016

**Authors:** Federico Batini, Giulia Toti, Marco Bartolucci

**Affiliations:** 1 University of Perugia, FISSUF, Piazza Ermini 1, 06123 Perugia, Italy.

**Keywords:** narrative, neuropsychology, dementia, cognitive training

## Abstract

Many areas in the cortex are active during reading of narrative material, and these activations in the brain produce significant changes in connectivity. Following previous results showing cognitive benefits in memory domains of a narrative training program in nursing home patients living with dementia, the aim of the present study was to perform a more in depth investigation of the effects of this training on memory domains and other cognitive areas.

**Methods:**

An experimental group of eight patients underwent auditory narrative training of 60 hours. At the beginning and end of the training, subjects were tested with a neuropsychological battery to quantify any improvements in individual performance.

**Results:**

The results showed a statistically significant improvement in the list learning task (immediate memory) and list learning recognition for single tasks, and a statistically significant improvement in overall cognitive area scores for immediate and delayed memory.

**Conclusion:**

Results replicate and expand our previous findings, indicating that this type of intervention can increase performance on memory-related tests.

## RESUMO

Muitas áreas no córtex são ativos durante a leitura do material narrativa, e essas ativações no cérebro podem produzir mudanças significativas na conectividade. Na sequência dos resultados anteriores mostrando os benefícios cognitivos em domínios de memória de um programa de treinamento narrativa em pacientes do lar de idosos que vivem com demência, o objetivo do presente estudo foi realizar uma investigação mais aprofundada dos efeitos desta formação sobre os domínios de memória e outras áreas cognitivas.

**Métodos:**

Um grupo experimental de oito pacientes foram submetidos a treinamento narrativa auditivo de 60 horas. No início e no final do treinamento, os participantes foram testados com uma bateria neuropsicológica para quantificar quaisquer melhorias no desempenho individual.

**Resultados:**

Os resultados mostraram uma melhoria estatisticamente significativa na tarefa lista de aprendizagem (memória imediata) e reconhecimento lista de aprendizagem para tarefas individuais e uma melhoria estatisticamente significativa na pontuação da área cognitivas gerais para a memória imediata e tardia.

**Conclusão:**

Replicar os resultados e expandir nossas descobertas anteriores, indicando que este tipo de intervenção pode aumentar o desempenho em testes relacionados à memória.

## INTRODUCTION

Research over the past two decades has shown many effects produced by reading in relation to cognitive domains. Reading and writing are activities that stimulate the brain and help to preserve memory. One study showed that the habit of engaging in mentally stimulating activities during the course of life strongly affects (in approximately 15%) the preservation of cognitive abilities during the aging process.^1^

Numerous studies in the literature have documented the positive effects of fiction reading for cognitive functions under normal or pathological conditions.^2^ ^,^ ^3^ The processing of an element of storytelling by the human brain is more complex than mere linguistic processing. This processing involves understanding the intentions, goals, emotions, and other mental states of the characters and is referred to as mentalizing.^4^ ^-^ ^8^

Many regions of the brain are involved in narrative understanding.^9^ Any network that supports the language, memory, and perception is likely to play a key role.^10^

A review of studies published in the literature has shown that the areas of the brain associated with reading are similar to those involved in autobiographical memory and the processes of mentalizing This correspondence has been interpreted as evidence that the process of mentalizing and of^11^ autobiographical memory are engaged during the processing of a story. 

In addition, autobiographical memory and the process of mentalizing (essential to understanding narrative) show similar patterns of activity in the medial ventrolateral prefrontal cortex, the precuneus, posterior cingulate, and in the retrosplenial cortex; into the medial-temporal and amygdala; and the temporo-parietal junction, along the superior temporal sulcus and the medium temporal gyrus at the temporal poles. In addition, there are evolutionary reasons for believing that the process of autobiographical memory and mentalizing are functionally related.^12^

During a task of understanding and/or the production of narrative material (from a functional point of view) many pre-frontal areas are active, involving the same areas recruited for recollecting and encoding of episodic and autobiographical memories.^13^ ^-^ ^18^ Moreover, a previous study has shown, in these areas, a detectable and significant common alteration in the resting state network (RSN) associated with reading. The authors found that reading novels can promote measurable changes in resting state connectivity of the brain in the left angular/supramarginal gyri and right posterior temporal gyri, regions previously associated with perspective taking and story comprehension, that decayed rapidly after the completion of the novel. In addition, other long-term changes in connectivity were observed, which persisted for several days after reading, in bilateral somatosensory cortex.^19^

In summary, many areas in the cortex are active during reading of narrative material and these activations in the brain produce significant changes in connectivity, and one can therefore speculate that reading can serve as efficient training not only for language, but all cognitive domains

The memory domains in individuals suffering from different forms of neurodegenerative diseases commonly called dementia, are the cognitive functions most affected. Impairments in episodic and autobiographical memory produce serious difficulties [20] (in parallel with the progression of the disease) and directly influence the quality of life of patients, undermining their sense of self (understood as awareness of ones´ subjectivity and integrity of personal history) and negatively impacting the everyday functioning of the individual.

The effectiveness of drug treatment in situations of pathological cognitive decline (dementia) has only proved effective for delaying the progression of symptoms. Therefore, non-pharmacological treatments become necessary to help elderly individuals address the functional and emotional consequences of cognitive decline. Ideally, these treatments should provide significant benefits in terms of improving or maintaining functional capacity through everyday activities and participation, enabling patients to achieve relevant personal goals, despite the increase in cognitive difficulties.

Thus, specific training that uses reading as a repeated stimulus should activate these areas, positively influencing the process of recovery of episodic and autobiographical memory. In patients who have preserved capacity at least for understanding the meaning of words and basic sentences, active listening to stories could serve as cognitive training for memory functions.^21^ Following these previous results, the aim of the present study was to carry out a more in-depth investigation of the possible cognitive outcomes of a literature-based intervention in patients affected by various forms of cognitive decline.

## METHODS

The experimental group consisted of eight subjects with a mean age of 82 years, including six women and two men, all patients living permanently in a nursing home. The sample was heterogeneous regarding degenerative diseases (Alzheimer's, Parkinson's disease, vascular dementia, etc.), psychiatric disorders, and degree of impairment/disease progression; thus, to extrapolate the experimental group, outcomes on the MMSE (mini-mental state examination) were used to evaluate the general cognitive decline status of all patients residing at the facility. This allowed us to create an experimental group on the basis of the severity of cognitive decline, as measured by the test (score of between 10 and 23 indicating a moderate level of cognitive impairment). In order to control influences other than the intervention, we analyzed MMSE [22] scores of the patients measured 18 months before training (at 18, 12 and 6 months prior, and at beginning training) and 6 months after training had finished (at the end and 6 months later)(see Results section). This was done to control whether activities patients were doing over the preceding 2 years at the nursing home had influenced their cognitive decline. No new activities were introduced into patients´ schedules during the course of the intervention.

The training was conducted with the assistance of a group of students from the University of Perugia (specially trained before beginning and supervised throughout the intervention) who read aloud to the experimental group for 60 sessions. The training took place on a daily basis, five days a week, Monday to Friday.

Subjects were seated (semicircle) in a room with no noise, in the presence of the readers and some educators (staff RSA) and listened to reading aloud. Listening time, initially limited (approximately 15-20 minutes), was gradually (over the course of about 20 meetings) increased to one hour (standard duration envisaged). The novels, initially short duration and low complexity, involved progressively longer and more complex texts.

Texts chosen initially were characterized by their overall brevity as well as the story period structure which was articulated in short sentences so that understanding was accessible even in situations of reduced memory span and consequent impairment of working memory. The texts used were characterized by progressively longer semantic units and greater total duration. Similarly, concerning the level of linguistic difficulty, the length of text was increased and texts not completed in a single day of reading were used, thus requiring patients to recollect contents of the previous episode.

The progressive comprehension difficulty and urge to remember, from one meeting to the next, were facilitated by the readers through the time provided for the patients to make comments and associations following the readings. The instructions given to readers were to facilitate and encourage this expression but not to promote it if not spontaneous. As an additional form of control for each session of reading, the reader had to note down everything that happened during the session in a logbook. The experimentation also incorporated, to assess adherence of the readers to the instructions received, numerous supervisory controls without prior notice.

The hypothesis underlying the construction of this type of training (still under development) is that the narrative acts as brain training in working memory domains with a progressively increasing scale of quantity and difficulty. The change in the final part of the overall training, where the effort required was no longer limited to a single meeting but extended to several meetings, was based on the assumption that the benefits gained in working memory during the first part of the training would be generalized to domains of long-term memory. In addition, most of the texts used were chosen on the basis of an alleged familiarity by the patients with authors and content.

At the beginning (baseline) and end of the training, patients were tested on specific neuropsychological domains to quantify any improvements in individual performance. The battery used was the RBANS.^23^ In addition, a qualitative test for autobiographical memory was used, where subjects were asked to recall an event in their lives. The examiner let the subject talk freely for three minutes by asking "tell me about a happy episode in your life;". In the post-training testing session, the examiner asked the same question. If the subject began recounting a different episode then the examiner tried to steer the subject toward the same episode told in the pre-training session using direct sentences reminding the individual about the exact episode (for example, "could you tell me something about your marriage....about when you were young" etc.). The registered three-minute countdown was then commenced. 

## RESULTS

First, the scores on the Mini-Mental State Examination performed by all the patients in the group from 18 months prior, up to the beginning of the training program (baseline) showed a clear negative trend of scores in all subjects, confirming that the other activities (none cognitively relevant) patients were doing over the preceding 18 months at the nursing home had no influence on their cognitive decline.

The averages of the raw scores on the neuropsychological tests were compared in the group of patients before and after training by within subject one-way ANOVA.

The results show a statistically significant improvement on the list learning task (immediate memory) (F(1,7) = 5.522 p < 0.05) and the list learning recognition task (delayed memory) (F(1,7) =11.796 p < 0.01).

Given that not all of the subjects in the experimental group completed the training with a decent number of sessions (all had some days when they could not participate in the reading session), results were also analysed only for those who attended at least 40 sessions (6 subjects).

Results of this sub-group revealed the same statistically significant improvement on the list learning task (immediate memory) (F(1,5) = 9.884, p < 0.01) and the list learning recognition task (delayed memory) (F(1,5) = 8.289 p < 0.01) seen for the whole group of participants, in addition to improvement on the story memory task (F(1,5) = 14.297 p < 0.01).

Finally, in the sub-group, a statistically significant improvement on the visuospatial task, the line orientation task (F(1,5) = 9.365 p < 0.01) was observed.

The RBANS allows analysis of data from single tasks by cognitive area, thus giving scores (by combining scores on single tasks pooled by cognitive area for which they are implemented) of five cognitive areas: immediate memory, visuospatial perception, language, attention, and delayed memory. Because the whole group and sub-group showed a positive trend for all the memory tasks performed alone, it was hypothesized that this could lead to statistically significant differences in the related cognitive area scores. As shown in the figures, both the groups had statistically significant differences in the areas of immediate memory (F(1,7) = 5.396 p < 0.05; F(1,5) = 19.073 p < 0.001) and delayed memory (F(1,7) = 6.087 p < 0.05; F(1,5) = 4.925 p < 0.05).

Although the sample was too small to statistically analyze the effects of the training in different pathologies, the effects were separated by sub-sample of subjects (2 Alzheimer's, 3 Parkinson's, 3 Vascular Dementia's and 1 Not specified) to qualitatively determine differences in improvement for immediate and delayed memory dimensions. As shown in Figure 7, although there appears to be an effect for all categories, this seems to be less relevant for the Alzheimer´s patients, albeit for immediate memory only.

**Figure 1:**
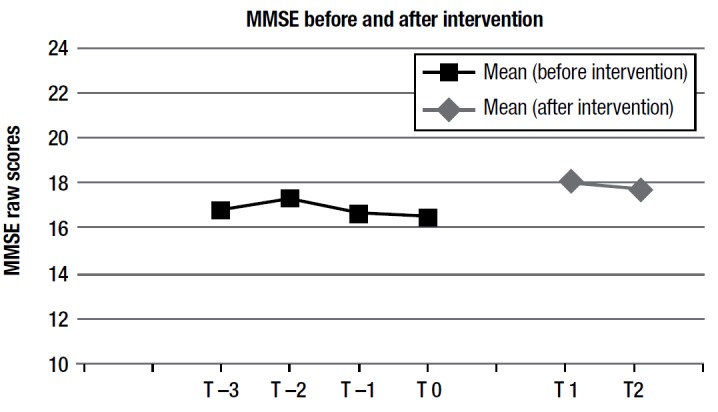
Mean MMSE scores at different time points: T-3 is 18 months before baseline, T-2 is 12 months before, T-1 is 6 months before, and T-0 is intervention baseline. T1 refers to scores at the end of the intervention and T2 at 6 months post-intervention.

**Figure 2:**
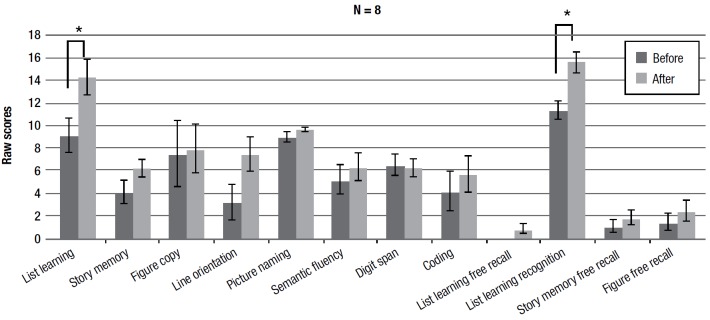
Pre- and post-training average scores on RBANS subtests.

**Figure 3:**
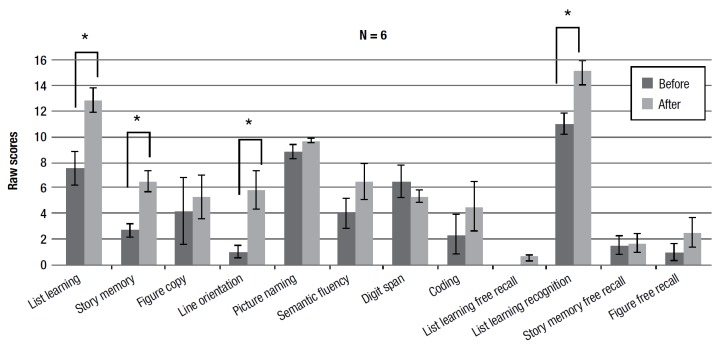
Pre- and post-training average scores on RBANS subtests for sub-group that completed at least 40 sessions of training.

**Figure 4:**
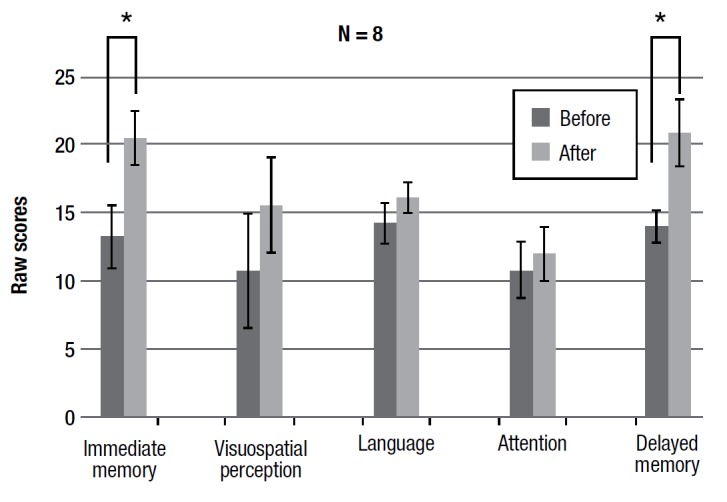
Pre- and post-training average scores for RBANS cognitive areas.

**Figure 5:**
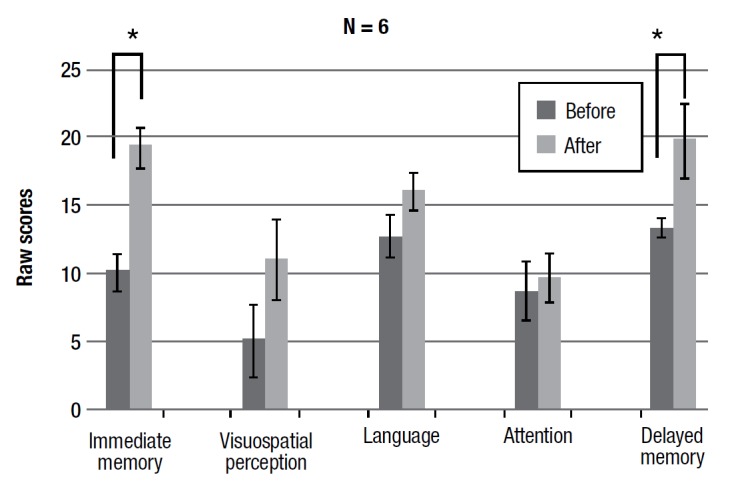
Pre- and post- training average scores of sub-group for RBANS cognitive areas.

**Figure 6:**
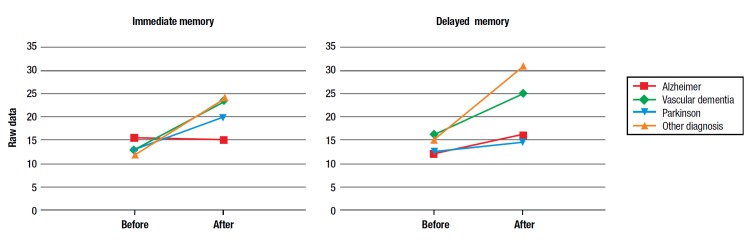
Pre- and post-training average scores by pathology for RBANS cognitive areas of Immediate and Delayed memory.

**Figure 7:**
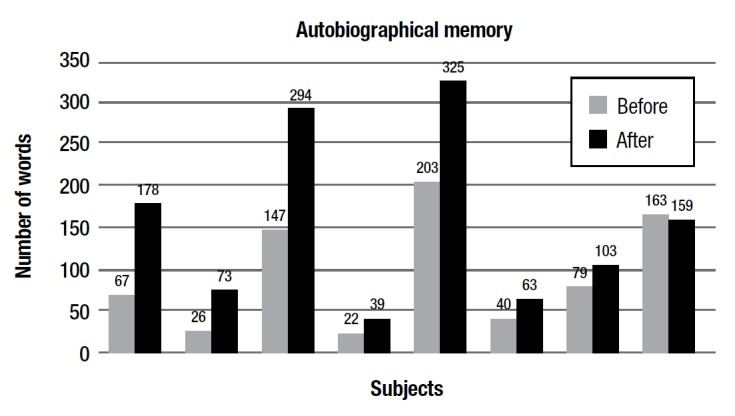
Pre- and post-training average number of words produced on autobiographical verbal production by subjects.

In addition, the difference between the average number of words produced in the autobiographical memory episode by the group was not statistically significant, although Fig. 2 shows a trend of increase for all subjects. In the first testing session, an average of 93,375 words were produced whereas in the second session (after the complete training) the average was 143,75, with an approximate 60% increase in words produced by the group (words produced when subject was recalling another episodic memory unrelated to the first session were excluded from the average of the second session).

The analysis of synoptic-semantic text revealed not only the significant length of production but also an increase in the complexity of the narrative structure of the story (some stories produced in the first session had a rather declarative structure according to the model, e.g.: a certain thing has happened to me in this place at this time) and enrichment of the emotional connotations.

A possible interpretation of these results is that the narrative training positively influenced long-term information storage (positive results on memory prose testing) by a re-learning effect to narratively organize memories and episodes, and also through stimulation of associative memory that the training may have promoted (at different stages of training patients reacted to readings in association with the events their own lives, recalling episodes, and associating other stories).

## DISCUSSION

The data indicate a clear improvement in the performances with respect to the domains of immediate and delayed memory. Although we used a different neuropsychological test in our previous study, some comparison of the results can be made. In this second study, we chose to change the tests (in the previous study we used story memory, digit span forward and backward, 3 objects in three places, all memory related tests) for two main reasons: first of all the RBANS is a neuropsychological battery that contains tests not only for memory domains but also attention, language and visuospatial perception, thus allowing for a more detailed investigation of any possible cognitive improvements. Second, RBANS has two equivalent forms enabling follow-up comparison.^24^ According to results of the previous study, a clear improvement effect for memory domains was obtained. It seems that actively listening to a story leads to more intense and deeper information processing. In order to understand the story (or single parts of it) we must put the verbal memory trace in contact with our experience and knowledge, our semantic categories, our images that we have in memory, our situation schemes, in a continuous dynamic interaction between what we hear and what we already have in our memory. Thus, this kind of "forced" training could even be generalized to learning of a list of single words. It can be assumed that training with narrative, that increases over time in complexity and duration, reinforces the ability to associate meanings with words. 

Surprisingly, little improvement was observed on the story retell sub-test (improvement is greater when only the 6 subjects who participated in at least 40 training sessions are considered). The other single tests that these subjects underwent between the story memory and the story recall test may have produced an interference effect. 

As previously stated, the training progressively increases in duration and difficulty of the narrative text read to the patient. Thus, results of the 6 subjects who attended at least 40 sessions also show a statistically significant increase in performance for episodic memory, data in line with previous results.^21^ Because both the whole group and the sub-group showed a positive trend for nearly all the tasks that involve memory, we analyzed data in a way that yields scores for the different cognitive areas. The RBANS allows analysis of data from the single tasks by cognitive area, thus giving scores (by combining scores of single tasks, pooled by cognitive area for which they are implemented) of 5 cognitive areas: immediate memory, visuospatial perception, language, attention, and delayed memory. For both the groups, there was a statistically significant difference in the domains of immediate and delayed memory. This suggests that the cognitive benefits of the training are not related to a single specific task, but improve the condition of the memory domain in general. The reinforcement in the ability to associate meanings with words can therefore be generalized to other mnestic material, ranging from single words to sentences and from verbal to visual material.

The learning effect could be another plausible explanation for the improvements that occurred on the neuropsychological tests. The scientific literature provides a large body of evidence supporting the effect of performance increases due to repeated exposure to specific tasks. The nature of the battery we used (repeatable) excluded this hypothesis. 

Also, before the intervention. patients were tested on a 6-monthly basis with the MMSE. Data showed a clear negative trend of scores for all subjects, indicating that the other activities (none cognitively relevant) patients were doing over the preceding 18 months at the nursing home were not influencing their cognitive decline. No new activities were introduced in patients´ time schedule during our intervention.

The understanding of stories, as highlighted in the literature, activates areas of the medial and dorsolateral prefrontal cortex,^24^ areas that are also active when a subject recalls or produces verbal episodic narrative material^25^ as well as autobiographical material. The data relating to the production of autobiographical material by the participants indicated enhanced potential for recollecting this material. One interpretation of these results is that listening training of narrative material stimulates narrative understanding and activates related areas, thus generalizing its effects to recollection of episodic and autobiographical material. In addition, listening to and understanding stories also activate a number of areas delegated to different cognitive functions, such as areas delegated to language, perception, attentional areas, motor areas and even visual areas;^26^ it could therefore be argued that this kind of training can generalize its benefits also to different cognitive domains and that this could explain the statistically significant difference on the visual orientation task. When we listen to a story we implicitly produce mental images in order to visualize the places, characters, and details described in the story within the brain. This kind of activity could thus serve as training for visual areas, producing positive effects on particular tasks.

Although the data described here are clear, they do not represent a specific relationship between the narrative training and a given pathology (forms of dementia such as Alzheimer´s or other types) at this stage. However, we provide a preliminary analysis of the scores before and after training for the two dimensions that presented significant changes, namely, immediate and delayed memory, stratified by different types of pathology. Results show some differences in the power of increase in performance, although it is too early to hypothesize on conclusions because the samples are too small. The nursing homes we work with are places of residence for people who require, as determined by a local hospital social worker and their nursing facility provider, continual nursing care and have significant difficulty coping with the basic activities of daily living. Thus, these facilities contain patients with different pathologies (here cognitive decline is the symptom of various diseases), degrees of cognitive impairment, and ages. The fact that these nursing homes can take only a few patients, leads to difficulty creating large experimental groups that are homogeneous for pathology and age.

In conclusion, all the data from this study points to the effectiveness of the daily narrative training in delaying cognitive decline and maybe in improving the overall quality of life and self-perception of the subjects. The idea that it might be possible to slow down cognitive decline by recovery and promotion of re-learning effects of cognitive performance in the pathological elderly population opens scenarios about the important contribution of narrative listening as a prevention tool. 
